# Global and Regional Associations of Smaller Cerebral Gray and White Matter Volumes with Gait in Older People

**DOI:** 10.1371/journal.pone.0084909

**Published:** 2014-01-08

**Authors:** Michele L. Callisaya, Richard Beare, Thanh G. Phan, Jian Chen, Velandai K. Srikanth

**Affiliations:** 1 Stroke and Ageing Research Group, Department of Medicine, Southern Clinical School, Monash University, Clayton, Victoria, Australia; 2 Menzies Research Institute Tasmania, University of Tasmania, Hobart, Tasmania, Australia; University Medical Center Rotterdam, Netherlands

## Abstract

**Background:**

Gait impairments increase with advancing age and can lead to falls and loss of independence. Brain atrophy also occurs in older age and may contribute to gait decline. We aimed to investigate global and regional relationships of cerebral gray and white matter volumes with gait speed, and its determinants step length and cadence, in older people.

**Methods:**

In a population-based study, participants aged >60 years without Parkinson's disease or brain infarcts underwent magnetic resonance imaging and gait measurements using a computerized walkway. Linear regression was used to study associations of total gray and white matter volumes with gait, adjusting for each other, age, sex, height and white matter hyperintensity volume. Other covariates considered in analyses included weight and vascular disease history. Voxel-based morphometry was used to study regional relationships of gray and white matter with gait.

**Results:**

There were 305 participants, mean age 71.4 (6.9) years, 54% male, mean gait speed 1.16 (0.22) m/s. Smaller total gray matter volume was independently associated with poorer gait speed (p = 0.001) and step length (p<0.001), but not cadence. Smaller volumes of cortical and subcortical gray matter in bilateral regions important for motor control, vision, perception and memory were independently associated with slower gait speed and shorter steps. No global or regional associations were observed between white matter volume and gait independent of gray matter volume, white matter hyperintensity volume and other covariates.

**Conclusion:**

Smaller gray matter volume in bilaterally distributed brain networks serving motor control was associated with slower gait speed and step length, but not cadence.

## Introduction

Gait speed has recently been identified as an important vital sign in aging individuals [1] due to its associations with falling, cognitive decline [2] and death [3]. Age may affect several characteristics of gait including speed and its determinants, step length and cadence [4]. Gait speed, step length and cadence can be easily measured in clinical settings and provide information as to why a person may have gait difficulty. Although slower gait may be influenced by age-related change in sensorimotor factors [5], [6], there is good evidence that it relies on central neural control [7]. Therefore gait may depend significantly on the integrity of the brain as one gets older.

A greater burden of cerebrovascular lesions, such as white matter hyperintensities (WMH) [8], [9], [10], [11], [12] and brain infarcts [9], [13] occur frequently in older age, and are associated with slower gait speed and step length. Cerebral gray and white matter also atrophy with age [14], [15], but their contribution to gait has received much less attention. Although smaller total brain volumes are associated with slower gait speed in healthy older people [8], there is limited knowledge regarding the regional effects of brain atrophy on gait. This issue has been examined only in a few studies [16], [17], [18], [19]. Reduced cortical thickness in prefrontal, supplementary motor, inferior parietal, cingulate and visual association areas was associated with poorer gait speed, step length and cadence in a clinical sample of patients with cerebrovascular disease [19]. In two other studies [16], [17], a region-of-interest approach was used to demonstrate associations of smaller volumes of dorsolateral prefrontal and parietal lobe gray matter with step length [16], and of left cerebellar and prefrontal gray matter with gait speed [17]. Although informative, these studies did not adopt a whole-brain approach and hence may have been limited in their ability to provide a comprehensive regional analysis. The use of voxel-based morphometry (VBM) can address this limitation by adopting an unbiased voxel-wise exploration of the whole brain to identify regional associations of gray and white matter volumes with gait.

The aim of this study was to identify global and regional associations of gray and white matter volume with gait speed, and its determinants step length and cadence. We chose these gait measures because it is of direct clinical interest (in diagnosing and treating gait disorders) whether slower gait speed is the result of shorter steps, slower cadence or a combination of both. We hypothesized that smaller volumes of gray and white matter in regions important for motor control would preferentially be associated with slower gait speed, smaller step length and slower cadence.

## Methods

### Ethics statement

The Southern Tasmanian Human Research Ethics Committee and the Monash University Human Research Ethics Committee approved the study and written informed consent was obtained from all participants. The study was conducted according to the principles expressed in the Declaration of Helsinki.

### Participants

We derived the sample from the Tasmanian Study of Cognition and Gait (TASCOG), a population-based study of brain aging. Tasmania is an island state of Australia and southern Tasmania has a total population of 239,444 people, including 46,159 aged >60 years [20]. Beginning in January 2005, we randomly selected residents between 60 and 86 years from the southern Tasmanian electoral roll. Participants were excluded if they lived in a nursing home or could not undergo magnetic resonance imaging (MRI), resulting in 395 eligible participants. For this analysis, we also excluded participants with MRI scans of insufficient quality (n = 17), those who were unable to walk without aids (n = 18) and those with a history of Parkinson's disease (n = 2) or a brain infarct on MRI (defined as hypointensity ≥3 mm in diameter on T1-weighted and FLAIR images, with a surrounding hyperintense rim on FLAIR) (n = 53) to avoid their confounding effects on gait. Those with a self-reported history of stroke but without MRI evidence of infarction (n = 16), were retained for this analysis.

### Gait Measures

We measured gait using the 4.6 metre *GAITRite* system (CIR Systems Inc), an electronic walkway with excellent validity [21]. Participants performed 6 walks at preferred pace and the following gait variables were averaged over the walks by the GaitRite software: speed, cadence and step length. Gait speed is the distance travelled divided by ambulation time (cm/sec); cadence is the number of steps taken per minute (steps/min); step length is the perpendicular distance between the heel of one footfall to the heel of the next footfall (cm). As there were no significant differences between left and right step lengths (p = 0.72), we used the average of both in further analyses.

### MRI

We obtained MRI scans using a single 1.5 T General Electric scanner with the following sequences: High-resolution T1 weighted spoiled gradient echo (TR35 ms, TE 7 ms, flip angle 35°, field of view 24 cm, 120 contiguous slices with images interpolated to give isotropic voxel size 1 mm^3^); T2 weighted fast spin echo (TR 4300 ms; TE 120 ms; NEX 1; turbo factor 48; voxel size 0.90×0.90×3 mm); FLAIR (TR = 8802 ms, TE = 130 ms, TI = 2200 ms, voxel size 0.50×0.50×3 mm); axial gradient echo (GRE, TR = 0.8 ms; TE = 0.015; flip angle 30°; voxel size = 0.9×0.9×7 mm).

### MRI Processing and Segmentation

Gray and white matter were segmented using both T1 and GRE sequences in a multispectral approach to avoid misclassification of deep gray matter structures that may occur if only T1 sequences were used. The higher contrast of these structures in GRE scans was used to correct their contrast in T1 weighted scans as follows: Linear transformations between T1, GRE and standard space were estimated using FMRIB's Linear Image Registration Tool [22]. An atlas of deep cortical structures was first transformed to T1 space and thresholding was performed using the intensity histogram segmentation method of Otsu [23] to remove cerebrospinal fluid. The resulting mask was transformed to GRE space followed by further thresholding to more accurately separate deep white and gray matter. The ensuing mask of deep gray matter was transformed back to T1 space and the gray matter regions were reset to 80% of the original brightness. The unified segmentation procedure implemented in the Statistical Parametric Mapping (SPM) software version 5 [24] were applied to this corrected T1 weighted scan. This software uses an expectation-maximization based approach to simultaneously classify each voxel of a T1 weighted scan, estimate a nonlinear transformation to standard Montreal Neurological Institute (MNI) space, and correct any MRI bias field inhomogeneities. The process produces standard space tissue probability maps of gray and white matter modulated by the transformation function to preserve tissue volume for each subject. We used previously validated semi-automated methods to segment WMH [10], [25]. Maps of normal appearing white matter were then created by marking locations corresponding to WMH as empty in the SPM-derived white matter probability maps. All tissue probability maps were smoothed using an isotropic Gaussian kernel, full width at half maximum  = 8 mm, prior to VBM analysis. Tissue volumes (total gray matter, white matter, WMH) were calculated by integrating the corrected tissue classification maps and applying voxel-counting algorithms.

### Other measurements

Height was obtained using a Leicester height stadiometer. Weight was obtained using a Heine Portable Professional Adult Scale 737. Blood pressure was measured using an Omron M4 sphygmomanometer in a sitting position. Physical activity levels were obtained using a Yamax Digi-Walker SW-200 pedometer worn over 7 consecutive days. Self-reported history of hypertension, hypercholesterolemia, ischemic heart disease, smoking history, diabetes mellitus, stroke, current psychoactive and blood-pressure lowering medications were obtained using a standardized questionnaire. Independence in activities of daily living was obtained using the Lawton's Instrumental Activities of Daily Living Scale (brief version) [26].

### Linear regression of total gray and white matter volume with gait measures

Multivariable linear regression was used to model the associations of total gray and normal appearing white matter volumes with each of the gait measures. Each model was first adjusted for age, sex and height (height being related to both head size and gait). Then, we additionally adjusted for vascular risk factors (weight, blood pressure, physical activity or self-reported vascular medical history) and medication use only if they changed the coefficient of the brain measure by more than 10% [27]. Finally, volumes of WMH, and then either normal appearing white matter (for gait regressions with gray matter), or gray matter (for gait regressions with normal appearing white matter) were added to the models. Statistical interaction between brain measures and other covariates was assessed by a test of the coefficient of the respective product terms (e.g. gray matter volume × sex, or gray matter volume × white matter volume). Non-linearity in associations was assessed by adding a term for the square of the relevant brain measure in the model and testing its significance. Analyses were conducted using STATA version 10.0 (StataCorp, College Station, TX).

### VBM analysis

VBM was used to determine the distributions of gray or white matter voxels that were associated with the gait measures based on multiple regression methods adopted in SPM software. In each model, we first adjusted for age, sex, and height. In our linear regression described in the previous section, we found that weight was a potential confounder only in the associations between white matter volume and gait. Therefore, in addition to age, sex and height, we adjusted for weight in VBM regressions of white matter volume. Finally in each model, adjustment was made accordingly for other brain measures. For example, regressions of gray matter with gait were adjusted for normal appearing white matter and vice versa, and both adjusted for WMH to remove any residual confounding by the latter. A false discovery rate of 0.05 was used to correct for multiple comparisons.

## Results

Characteristics of the sample are provided in Table 1. The mean age of the sample was 71.4 (6.9) years, 54% were male and the mean gait speed was 1.16 (0.22) m/s. The sample response rate was 55% (395/719). Non-responders were more likely to be older and report a history of hypertension (both p<0.001). Compared with the full sample, those excluded for the presence of MRI brain infarcts were older (p<0.001), walked with poorer gait speed, cadence and step length (all p<0.05) and had smaller volumes of gray (p = 0.002) and white matter (p = 0.02).

**Table 1:** Characteristics of Participants in the TASCOG (n = 305).

Age, mean (SD)	71.4	(6.9)
Males, n (%)	165	(54.1)
Height (cm)	166.9	(9.3)
Weight (kg)	77.1	(14.3)
Diastolic blood pressure (mmHg)	142.9	(22.3)
Systolic blood pressure (mmHg)	80.9	(12.7)
Independent in activities of daily living (%)*	291	(95.4)
Physical activity (steps per day), mean (SD)	6293.3	3171.8
Self-reported medical history		
Hypertension, n (%)	145	(47.5)
Diabetes, n (%)	26	(8.5)
Ischemic heart disease, n (%)	35	(11.5)
Hypercholesterolemia, n (%)	116	(38.0)
Stroke	16	(5.3)
Ever smoker, n (%)	152	(49.8)
Arthritis, n (%)	128	(42.2)
Psychoactive medication, n (%)	51	16.6%
Blood pressure lowering medication, n (%)	149	48.9%
Gait measures, mean (SD)		
Gait speed, cm/sec	1.16	(0.22)
Cadence, steps/min	110.8	(10.9)
Step length, cm	62.5	(9.2)
Brain volume (ml), mean (SD)		
Total brain volume	1437.8	(141.3)
Gray matter volume	483.6	(62.2)
White matter volume	572.5	(64.6)
WMH volume	10.9	(7.7)

Lawton's Instrumental Activities of Daily Living Scale ≥22/24; SD = standard deviation; WMH = white matter hyperintensities.

### Linear regression of total brain gray and white matter volumes with gait measures

After adjustment for age, sex and height, smaller total gray matter volume was associated with slower gait speed (β 0.08, 95% CI 0.04, 0.13; p<0.001) and smaller step length (β 0.04, 95% CI 0.02, 0.05; p<0.001), but not with cadence (β 0.02, 95%CI −0.01,0.04; p = 0.12). Adjustment for weight, blood pressure, physical activity or vascular history (hypertension, hypercholesterolemia, ischemic heart disease, smoking history, diabetes mellitus, and stroke) and current psychoactive or blood-pressure lowering medications did not alter the associations and were therefore not included in the models. The addition of WMH volume and normal appearing white matter volume only minimally altered the associations (Table 2).

**Table 2:** Associations between gray and white matter volume and gait measures adjusted for covariates (n = 305).

*Gray matter volume, ml*							*White matter volume, ml*					
	Model 1 Adjusted for age, sex and height			Model 2 Additionally adjusted for WMH and normal appearing white matter volume			Model 1 Adjusted for age, sex, height and weight			Model 2 Additionally adjusted for WMH and gray matter volume		
*Gait variable*	β	(95% CI)	P value	β	(95% CI)	P value	β	(95% CI)	P value	β	(95% CI)	P value
Gait speed (cm/sec)	0.08	(0.04,0.13)	<0.001	0.08	(0.03, 0.13)	0.001	0.04	(0.00, 0.08)	0.03	0.04	(−0.0,0.08)	0.07
Step length (cm)	0.04	(0.02,0.05)	<0.001	0.04	(0.02, 0.05)	<0.001	0.02	(0.00, 0.03)	0.03	0.01	(−0.00,0.02)	0.06
Cadence (steps/min)	0.02	(−0.01,0.04)	0.12	0.02	(−0.06, 0.04)	0.15	0.01	(−0.01, 0.03)	0.27	0.01	(−0.01, 0.03)	0.35

WMH = white matter hyperintensities; β = beta coefficient; CI  =  confidence intervals.

Total normal appearing white matter volume was not associated with gait speed (β 0.03, 95%CI −0.01, 0.07; p = 0.09), step length (β 0.01, 95%CI −0.00, 0.03; p = 0.11) or cadence (β 0.01, 95%CI −0.01, 0.03; p = 0.33) after adjustment for age, sex and height. After additional adjustment for weight, smaller normal appearing white matter volume was associated with slower gait speed (β 0.04 95% CI 0.00, 0.08; p = 0.03) and step length (β 0.02 95%CI 0.00, 0.03; p = 0.03), but not with cadence (β 0.01 95% CI−0.01, 0.03; p = 0.27). Adjustment for blood pressure, medications, physical activity or medical history did not alter the associations and were therefore not included in the models. However, further adjustment for the gray matter and WMH volumes rendered all associations for white matter volume non-significant (Table 2).

All associations for gray and white matter volumes were linear and no interactions were observed between covariates.

### VBM of gray matter with gait

**Figure 1:**
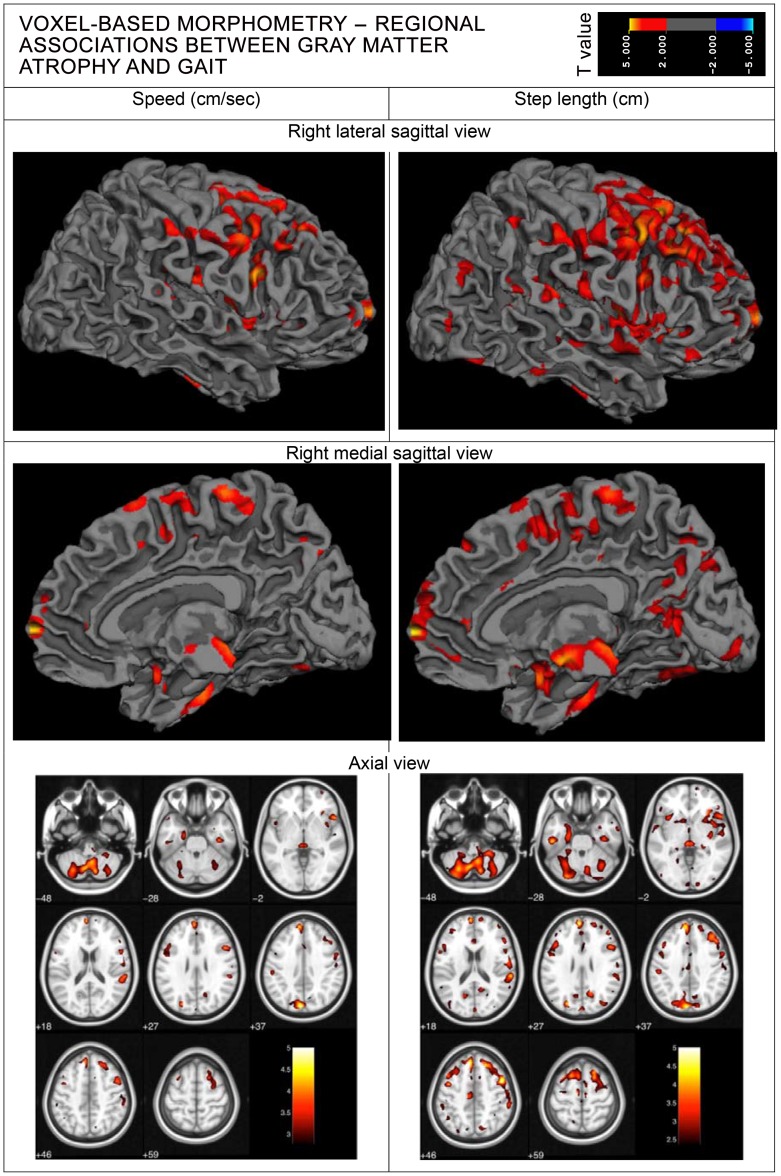
Voxel-based morphometry - regional associations between gray matter atrophy and gait.

After covarying for age, sex and height, smaller gray matter volume in bilateral frontal, cingulate, insula, temporal, parahippocampal, parietal, occipital and cerebellar areas were associated with slower gait and shorter steps. Smaller volumes of subcortical gray matter in the thalamus, caudate nucleus, putamen and claustrum were also similarly associated with these gait measures. Adjustment for normal appearing white matter removed the associations of gray matter with gait speed in the right precuneus, left caudate nucleus, left thalamus, left inferior occipital and left inferior temporal lobes. Further adjustment for WMH removed the associations in the right superior parietal and right inferior occipital lobes, right cuneus, right insula and left anterior cingulate areas. Associations with step length were only slightly attenuated in these areas, but all remained (Figure 1). No regional associations were found between gray matter volume and cadence. Table S1 (supporting information) provides Talairach coordinates for peak associations of smaller gray matter volumes with gait speed and step length, the relevant structures involved, and their principal functions that may influence gait. Step length remained associated with smaller gray matter volume in frontal, cingulate, insula, temporal, parahippocampal, parietal, occipital and cerebellar areas, as well as the thalamus, caudate nucleus, putamen and the claustrum. Gait speed was associated with similar areas of gray matter except that areas tended to be smaller and no associations were found in the superior parietal lobe or caudate nucleus.

### VBM of white matter with gait

After adjusting for age, sex, height and weight, smaller volumes of normal appearing white matter in corticospinal tracts, forceps major, forceps minor, inferior fronto-occipital fasciculus, inferior and superior longitudinal fasciculus, uncinate fasciculus, cingulum and the anterior thalamic radiation were associated with slower gait speed and shorter steps. However, all these associations disappeared when gray matter and WMH volume were added to the models as covariates.

## Discussion

This study provides novel voxel-based data on the regional associations of gray and white matter volume with gait in older people. Bilateral and largely symmetrical regions of smaller gray matter volume in areas relevant to motor control were associated with slower gait speed and shorter steps, but not cadence. Some of the associations between regional gray matter volumes and gait may be explained by reduced volumes of normal appearing white matter or the presence of WMH. After controlling for covariates including gray matter and WMH volumes, normal appearing white matter was not independently associated with any of the gait measures.

These results are consistent with the notion that gait requires the complex interaction of distributed brain cortical networks [7], [28] important for initiating (basal ganglia, supplementary motor area), regulating (basal ganglia) and executing movement (pre-motor, supplementary and primary motor areas), somatosensory function (parietal cortex, precuneus [29]), relaying sensory information (thalamus), visual processing (occipital cortex, cuneus) and balance (precentral and prefrontal cortex, cerebellum, subcortical nuclei). They are also consistent with the understanding that areas serving attention and cognitive control (prefrontal, insula, temporal lobe, subcortical nuclei, limbic structures) [30], [31], [32], [33] are important for regulating gait. These findings add substantially to evidence from previous region-of-interest studies [16], [17], [18], in which fewer regional associations were reported for gait speed and step length, namely in the cerebellum, prefrontal, motor and parietal cortices. In contrast to a previous study in patients with cerebral small vessel disease, we found that gray matter was unrelated to cadence [19]. This difference in results may be explained by the fact that we used a population-based sample and excluded those with MRI brain infarct. Alternatively cadence may have a different neurological basis compared with step length [34]. In support of this view, others that have suggested that cadence may be influenced more by the brainstem and spinal cord, rather than the cerebral cortex [35], [36]. Although smaller white matter volumes involving major frontal projection and association white matter tracts were related to poorer gait speed and step length these associations were completely accounted for by the variations in gray matter or WMH volumes. Therefore, they may reflect the distribution of gray matter regions connected by these tracts, or alternatively the disconnection of these tracts by WMH.

There may be several mechanisms underlying brain atrophy in older age [37]. Infarcts, WMH, vascular and lifestyle risk factors [38], [39] have been associated with brain atrophy. The observed associations between gray matter volume and gait in our study were independent of these factors, suggesting that primary neurodegenerative mechanisms may be more likely at play. Neuronal apoptosis, oxidative stress and inflammation [40], or other pathological processes such as beta-amyloid and tau accumulation [41], are all known to contribute to neurodegeneration in older age. However, given that we only had self-reported measures of cardiovascular history, we cannot completely discount the possibility that unmeasured cardiovascular factors such as arterial stiffness [42] may explain some of the observed associations. Understanding the underlying mechanisms of age-related brain atrophy will be important in order to preserve gait in old age, particularly when gait becomes more reliant on cognitive control to compensate for decline in peripheral sensorimotor function [43]. Physical and cognitive activity, as well as controlling mid-life vascular risk may assist in preventing gray matter loss [38], [44], with the consequent potential to preserve walking speed and step length in later life.

Our study has the following strengths. We obtained sensitive and accurate measurements of brain structure and gait. We used VBM in a large sample to provide an unbiased voxel-wise regional exploration of the whole brain while correcting carefully for multiple comparisons. While studying the associations of either gray or white matter with gait, we controlled for one or the other, thus being able to draw conclusions about their relative effects. There are also limitations to this study. The cross-sectional design does not allow causal inferences to be made, with the possibility that slower gait may precede brain atrophy, although longitudinal follow-up in our cohort may shed more light on this matter. We excluded people with self-reported prior Parkinson's disease, and hence the potential exists for the presence of undetected or subclinical Parkinson's disease in the sample which may have confounded the association between brain measures and gait. But the likelihood for this is extremely small given its low prevalence in the general population. Although we conducted a population-based study, our exclusion criteria may have resulted in a relatively healthy sample, thus limiting generalizability to all community-dwelling older people. We did not include measures of gait variability which may be more sensitive markers of falls risk in older people [45]. Lastly, without more advanced MRI techniques such as diffusion tensor imaging, we were unable to study the relationship between the functional integrity of white matter and gait, the former suggested as a more sensitive marker of white matter injury than just volume [12].

## Conclusion

Smaller gray matter volumes in bilaterally distributed cortical and subcortical regions were independently associated with slower gait speed and step length, partly explained by reduced white matter volume or the presence of WMH. Longitudinal follow-up of this cohort may shed further light on the causality of the relationships between regional gray matter atrophy and gait decline.

## Supporting Information

Table S1 Regional Correlates of gray matter atrophy with gait [Talairach Atlas coordinates and Brodmann areas (BA)].
(DOCX)

## References

[1] Cesari M (2011) Role of gait speed in the assessment of older patients. JAMA 305: 93–94. 21205972 10.1001/jama.2010.1970

[2] Verghese J, Wang C, Holtzer R, Lipton R, Xue X (2007) Quantitative gait dysfunction and risk of cognitive decline and dementia. J Neurol Neurosurg Psychiatry 78: 929–935. 17237140 10.1136/jnnp.2006.106914 PMC1995159

[3] Montero-Odasso M, Schapira M, Soriano ER, Varela M, Kaplan R, et al (2005) Gait velocity as a single predictor of adverse events in healthy seniors aged 75 years and older. J Gerontol A Biol Sci Med Sci 60: 1304–1309. 16282564 10.1093/gerona/60.10.1304

[4] Callisaya ML, Blizzard L, Schmidt MD, McGinley JL, Srikanth VK (2008) Sex modifies the relationship between age and gait: a population-based study of older adults. J Gerontol A Biol Sci Med Sci 63: 165–170. 18314452 10.1093/gerona/63.2.165

[5] Lord SR, Lloyd DG, Li SK (1996) Sensori-motor function, gait patterns and falls in community-dwelling women. Age Ageing 25: 292–299. 8831874 10.1093/ageing/25.4.292

[6] Callisaya ML, Blizzard L, Schmidt MD, McGinley JL, Lord SR, et al (2009) A population-based study of sensorimotor factors affecting gait in older people. Age Ageing 38: 290–295. 19264860 10.1093/ageing/afp017

[7] la Fougere C, Zwergal A, Rominger A, Forster S, Fesl G, et al (2010) Real versus imagined locomotion: a [18F]-FDG PET-fMRI comparison. Neuroimage 50: 1589–1598. 20034578 10.1016/j.neuroimage.2009.12.060

[8] Rosano C, Sigurdsson S, Siggeirsdottir K, Phillips CL, Garcia M, et al (2010) Magnetization transfer imaging, white matter hyperintensities, brain atrophy and slower gait in older men and women. Neurobiol Aging 31: 1197–1204. 18774624 10.1016/j.neurobiolaging.2008.08.004 PMC2873052

[9] Rosano C, Brach J, Longstreth WT Jr, Newman AB (2006) Quantitative measures of gait characteristics indicate prevalence of underlying subclinical structural brain abnormalities in high-functioning older adults. Neuroepidemiology 26: 52–60. 16254454 10.1159/000089240

[10] Srikanth V, Beare R, Blizzard L, Phan T, Stapleton J, et al (2009) Cerebral white matter lesions, gait, and the risk of incident falls: a prospective population-based study. Stroke 40: 175–180. 18927448 10.1161/STROKEAHA.108.524355

[11] Baezner H, Blahak C, Poggesi A, Pantoni L, Inzitari D, et al (2008) Association of gait and balance disorders with age-related white matter changes: the LADIS study. Neurology 70: 935–942. 18347315 10.1212/01.wnl.0000305959.46197.e6

[12] de Laat KF, Tuladhar AM, van Norden AG, Norris DG, Zwiers MP, et al (2011) Loss of white matter integrity is associated with gait disorders in cerebral small vessel disease. Brain 134: 73–83. 21156660 10.1093/brain/awq343

[13] de Laat KF, van Norden AG, Gons RA, van Oudheusden LJ, van Uden IW, et al (2010) Gait in elderly with cerebral small vessel disease. Stroke 41: 1652–1658. 20576951 10.1161/STROKEAHA.110.583229

[14] Raji CA, Lopez OL, Kuller LH, Carmichael OT, Becker JT (2009) Age, Alzheimer disease, and brain structure. Neurology 73: 1899–1905. 19846828 10.1212/WNL.0b013e3181c3f293 PMC2788799

[15] Galluzzi S, Beltramello A, Filippi M, Frisoni GB (2008) Aging. Neurol Sci 29 Suppl 3: 296–300. 18941717 10.1007/s10072-008-1002-6

[16] Rosano C, Aizenstein H, Brach J, Longenberger A, Studenski S, et al (2008) Special article: gait measures indicate underlying focal gray matter atrophy in the brain of older adults. J Gerontol A Biol Sci Med Sci 63: 1380–1388. 19126852 10.1093/gerona/63.12.1380 PMC2648808

[17] Rosano C, Aizenstein HJ, Studenski S, Newman AB (2007) A regions-of-interest volumetric analysis of mobility limitations in community-dwelling older adults. J Gerontol A Biol Sci Med Sci 62: 1048–1055. 17895446 10.1093/gerona/62.9.1048

[18] Rosano C, Studenski SA, Aizenstein HJ, Boudreau RM, Longstreth WT Jr, et al (2012) Slower gait, slower information processing and smaller prefrontal area in older adults. Age Ageing 41: 58–64. 21965414 10.1093/ageing/afr113 PMC3234076

[19] de Laat KF, Reid AT, Grim DC, Evans AC, Kotter R, et al (2011) Cortical thickness is associated with gait disturbances in cerebral small vessel disease. Neuroimage 59: 1478–1484. 21854857 10.1016/j.neuroimage.2011.08.005

[20] Australian Bureau of Statistics (2005) Population by age and sex, Tasmania [data cube: Super Table online]. In: Statistics ABo, editor. ABS publication.

[21] Webster KE, Wittwer JE, Feller JA (2005) Validity of the GAITRite walkway system for the measurement of averaged and individual step parameters of gait. Gait Posture 22: 317–321. 16274913 10.1016/j.gaitpost.2004.10.005

[22] Jenkinson M, Smith S (2001) A global optimisation method for robust affine registration of brain images. Med Image Anal 5: 143–156. 11516708 10.1016/s1361-8415(01)00036-6

[23] Otsu N (1979) A Threshold Selection Method from Gray-Level Histograms. EEE Transactions on Systems, Man and Cybernetics 9: 62–66.

[24] Ashburner J, Friston KJ (2005) Unified segmentation. Neuroimage 26: 839–851. 15955494 10.1016/j.neuroimage.2005.02.018

[25] Beare R, Srikanth V, Chen J, Phan TG, Stapleton J, et al (2009) Development and validation of morphological segmentation of age-related cerebral white matter hyperintensities. Neuroimage 47: 199–203. 19344777 10.1016/j.neuroimage.2009.03.055

[26] Lawton MP, Brody EM (1969) Assessment of older people: self-maintaining and instrumental activities of daily living. Gerontologist 9: 179–186. 5349366

[27] Maldonado G, Greenland S (1993) Simulation study of confounder-selection strategies. Am J Epidemiol 138: 923–936. 8256780 10.1093/oxfordjournals.aje.a116813

[28] Wang C, Wai Y, Kuo B, Yeh YY, Wang J (2008) Cortical control of gait in healthy humans: an fMRI study. J Neural Transm 115: 1149–1158. 18506392 10.1007/s00702-008-0058-z

[29] Margulies DS, Vincent JL, Kelly C, Lohmann G, Uddin LQ, et al (2009) Precuneus shares intrinsic functional architecture in humans and monkeys. Proc Natl Acad Sci U S A 106: 20069–20074. 19903877 10.1073/pnas.0905314106 PMC2775700

[30] Paus T (2001) Primate anterior cingulate cortex: where motor control, drive and cognition interface. Nat Rev Neurosci 2: 417–424. 11389475 10.1038/35077500

[31] Srikanth V, Sanders L, Callisaya M, Martin K, Phan T (2010) Brain aging and gait. Aging Health 6: 123–131.

[32] Martin KL, Blizzard L, Wood AG, Srikanth V, Thomson R, et al (2012) Cognitive Function, Gait, and Gait Variability in Older People: A Population-Based Study. J Gerontol A Biol Sci Med Sci 68: 726–732. 23112113 10.1093/gerona/gls224

[33] Holtzer R, Verghese J, Xue X, Lipton RB (2006) Cognitive processes related to gait velocity: results from the Einstein Aging Study. Neuropsychology 20: 215–223. 16594782 10.1037/0894-4105.20.2.215

[34] Hollman JH, McDade EM, Petersen RC (2011) Normative spatiotemporal gait parameters in older adults. Gait Posture 34: 111–118. 21531139 10.1016/j.gaitpost.2011.03.024 PMC3104090

[35] Dietz V (2003) Spinal cord pattern generators for locomotion. Clin Neurophysiol 114: 1379–1389. 12888019 10.1016/s1388-2457(03)00120-2

[36] Al-Yahya E, Dawes H, Smith L, Dennis A, Howells K, et al (2011) Cognitive motor interference while walking: a systematic review and meta-analysis. Neurosci Biobehav Rev 35: 715–728. 20833198 10.1016/j.neubiorev.2010.08.008

[37] Ikram MA, Vrooman HA, Vernooij MW, van der Lijn F, Hofman A, et al (2008) Brain tissue volumes in the general elderly population. The Rotterdam Scan Study. Neurobiol Aging 29: 882–890. 17239994 10.1016/j.neurobiolaging.2006.12.012

[38] Leritz EC, Salat DH, Williams VJ, Schnyer DM, Rudolph JL, et al (2011) Thickness of the human cerebral cortex is associated with metrics of cerebrovascular health in a normative sample of community dwelling older adults. Neuroimage 54: 2659–2671. 21035552 10.1016/j.neuroimage.2010.10.050 PMC3026290

[39] Meyer JS, Terayama Y, Konno S, Akiyama H, Margishvili GM, et al (1998) Risk factors for cerebral degenerative changes and dementia. Eur Neurol 39 Suppl 1: 7–16. 9516069 10.1159/000052064

[40] Hampel H, Burger K, Teipel SJ, Bokde AL, Zetterberg H, et al (2008) Core candidate neurochemical and imaging biomarkers of Alzheimer's disease. Alzheimers Dement 4: 38–48. 18631949 10.1016/j.jalz.2007.08.006

[41] Fjell AM, Walhovd KB, Fennema-Notestine C, McEvoy LK, Hagler DJ, et al (2010) Brain atrophy in healthy aging is related to CSF levels of Abeta1-42. Cereb Cortex 20: 2069–2079. 20051356 10.1093/cercor/bhp279 PMC3025722

[42] Mitchell GF (2008) Effects of central arterial aging on the structure and function of the peripheral vasculature: implications for end-organ damage. J Appl Physiol (1985) 105: 1652–1660. 18772322 10.1152/japplphysiol.90549.2008 PMC2584844

[43] Seidler RD, Bernard JA, Burutolu TB, Fling BW, Gordon MT, et al (2010) Motor control and aging: links to age-related brain structural, functional, and biochemical effects. Neurosci Biobehav Rev 34: 721–733. 19850077 10.1016/j.neubiorev.2009.10.005 PMC2838968

[44] Colcombe SJ, Erickson KI, Scalf PE, Kim JS, Prakash R, et al (2006) Aerobic exercise training increases brain volume in aging humans. J Gerontol A Biol Sci Med Sci 61: 1166–1170. 17167157 10.1093/gerona/61.11.1166

[45] Hausdorff JM, Rios DA, Edelberg HK (2001) Gait variability and fall risk in community-living older adults: a 1-year prospective study. Arch Phys Med Rehabil 82: 1050–1056. 11494184 10.1053/apmr.2001.24893

