# Supplementary material for: Global and Regional Associations of Smaller Cerebral Gray and White Matter Volumes with Gait in Older People
Source: PLoS One. 2014 Jan 8;9(1):e84909. doi: 10.1371/journal.pone.0084909 (PMC3885624; doi:10.1371/journal.pone.0084909)
Supplement: Table S1 — Regional Correlates of gray matter atrophy with gait [Talairach Atlas coordinates and Brodmann areas (BA)]. (DOCX) [file pone.0084909.s001.docx]

| Table S1: Regional Correlates of gray matter atrophy with gait [Talairach Atlas coordinates and Brodmann areas (BA)] | | | | | | | | | | | | |  |
| --- | --- | --- | --- | --- | --- | --- | --- | --- | --- | --- | --- | --- | --- |
|  | | | | | | | | | | | | |  |
|  |  | **GAIT SPEED** | | | | | **STEP LENGTH** | | | | |  | |
| **Location** | **H** | **Coordinates** | | | **BA** | **T value^*^** | **Coordinates** | | | **BA** | **T value^*^** | **Principal function served**^†^ | |
| **Frontal lobe** |  |  |  |  |  |  |  |  |  |  |  | Motor function -planning and execution; Executive function and attention | |
| Precentral gyrus | Left | -45 | 0 | 31 | 6 | 3.42 | -43 | -4 | 41 | 6 | 3.69 |  | |
|  |  |  |  |  |  |  |  |  |  |  |  |  | |
|  | Right | 49 | -2 | 38 | 6 | 3.52 | 56 | -4 | 8 | 6 | 2.91 |  | |
|  |  | 49 | -16 | 41 | 4 | 3.40 |  |  |  |  |  |  | |
| Superior frontal gyrus | Left | -6 | 51 | 29 | 9 | 3.96 | -19 | 58 | 18 | 10 | 2.56 |  | |
|  |  | -6 | 38 | 45 | 8 | 4.06 | -6 | 53 | 22 | 9 | 3.64 |  | |
|  |  | -17 | 17 | 53 | 6 | 2.93 | -4 | 37 | 43 | 8 | 3.83 |  | |
|  |  |  |  |  |  |  | -22 | 11 | 55 | 6 | 3.60 |  | |
|  | Right | 9 | 54 | 25 | 9 | 3.11 | 17 | 30 | 48 | 8 | 3.41 |  | |
|  |  | 24 | 25 | 48 | 8 | 3.65 | 17 | 22 | 53 | 6 | 3.25 |  | |
|  |  | 6 | 7 | 63 | 6 | 3.16 |  |  |  |  |  |  | |
| Middle frontal gyrus | Left | -43 | 13 | 29 | 9 | 3.41 | -41 | 15 | 31 | 9 | 3.84 |  | |
|  |  | -26 | 14 | 53 | 6 | 3.33 | -28 | 18 | 47 | 8 | 3.11 |  | |
|  |  |  |  |  |  |  | -23 | -7 | 48 | 6 | 2.60 |  | |
|  | Right | 41 | 0 | 47 | 6 | 3.87 | 41 | 0 | 45 | 6 | 4.75 |  | |
|  |  |  |  |  |  |  | 36 | 44 | 27 | 9 | 2.65 |  | |
|  |  |  |  |  |  |  |  |  |  |  |  |  | |
| Medial frontal gyrus | Left | -5 | 51 | -3 | 10 | 3.18 | -3 | 36 | -11 | 11 | 2.76 |  | |
|  |  | -3 | 45 | 35 | 6 | 3.75 | -3 | 54 | 16 | 9 | 3.86 |  | |
|  |  | -3 | 53 | 16 | 9 | 4.26 | -4 | 46 | 34 | 6 | 4.49 |  | |
|  |  |  |  |  |  |  |  |  |  |  |  |  | |
|  | Right | 7 | 58 | 13 | 10 | 4.23 | 9 | 47 | 3 | 10 | 2.62 |  | |
|  |  | 4 | -6 | 48 | 6 | 3.30 | 5 | -7 | 51 | 6 | 3.59 |  | |
| Inferior frontal gyrus | Left | -54 | 4 | 17 | 44 | 3.23 | -43 | 19 | -14 | 47 | 2.46 |  | |
|  |  | -44 | 11 | 29 | 9 | 3.30 | -54 | 4 | 16 | 44 | 2.93 |  | |
|  | Right | 46 | 10 | 29 | 9 | 4.26 | 40 | 24 | 2 | 47 | 2.51 |  | |
|  |  |  |  |  |  |  | 45 | 10 | 27 | 9 | 3.92 |  | |
| **Parietal lobe** |  |  |  |  |  |  |  |  |  |  |  | Sensation and perception; Visuospatial and visuomotor processing | |
| Post central gyrus | Left | -50 | -29 | 36 | 2 | 3.50 | -50 | -29 | 36 | 2 | 3.34 |  | |
|  |  |  |  |  |  |  |  |  |  |  |  |  | |
|  | Right | 53 | -28 | 42 | 2 | 3.23 | 54 | -20 | 30 | 2 | 2.97 |  | |
|  |  |  |  |  |  |  | 50 | -19 | 41 | 3 | 2.96 |  | |
| Superior parietal lobe | Left |  |  |  |  |  | -18 | -55 | 59 | 7 | 2.64 |  | |
|  | Right |  |  |  |  |  | 12 | -57 | 59 | 7 | 2.47 |  | |
| Inferior parietal lobe | Left | -52 | -36 | 41 | 40 | 2.97 | -51 | -37 | 41 | 40 | 2.75 |  | |
|  | Right |  |  |  |  |  | 52 | -37 | 43 | 40 | 3.47 |  | |
| Precuneus | Left | -11 | -62 | 32 | 7 | 2.80 | -9 | -61 | 32 | 7 | 2.87 |  | |
|  | Right |  |  |  |  |  | 40 | -65 | 37 | 39 | 2.64 |  | |
|  |  |  |  |  |  |  | 3 | -76 | 45 | 7 | 2.37 |  | |
| **Temporal lobe** |  |  |  |  |  |  |  |  |  |  |  | Language; Memory; Navigation | |
| Superior temporal gyrus | Left | -44 | 17 | -19 | 38 | 3.16 | -45 | 18 | -21 | 38 | 2.37 |  | |
|  |  |  |  |  |  |  | -53 | -47 | 10 | 33 | 2.70 |  | |
|  | Right | 49 | -8 | 1 | 22 | 2.95 | 47 | 8 | -18 | 38 | 2.66 |  | |
|  |  |  |  |  |  |  | 48 | -9 | 0 | 22 | 3.15 |  | |
| Middle temporal gyrus | Left | -46 | 7 | -28 | 21 | 2.78 | -45 | 8 | -28 | 21 | 2.80 |  | |
|  |  |  |  |  |  |  | -53 | -58 | -10 | 37 | 2.60 |  | |
|  | Right | 54 | -1 | -22 | 21 | 2.77 | 50 | -57 | 10 | 39 | 3.09 |  | |
| Inferior temporal gyrus | Left |  |  |  |  |  | -57 | -29 | -16 | 20 | 2.62 |  | |
|  |  |  |  |  |  |  | -54 | -57 | -10 | 37 | 2.65 |  | |
|  | Right | 53 | -39 | -17 | 37 | 2.80 | 50 | -38 | -17 | 37 | 2.87 |  | |
|  |  |  |  |  |  |  |  |  |  |  |  |  | |
| Fusiform | Left | -39 | -19 | -23 | 20 | 2.86 | -46 | -21 | -24 | 20 | 4.18 |  | |
|  |  |  |  |  |  |  | -46 | -40 | -19 | 36 | 3.49 |  | |
|  | Right | 37 | -19 | -25 | 20 | 3.06 | 39 | -17 | -25 | 20 | 2.99 |  | |
| **Occipital lobe** |  |  |  |  |  |  |  |  |  |  |  | Visual/spatial processing | |
| Middle occipital gyrus | Left | -41 | -81 | 7 | 19 | 2.92 | -45 | -75 | -6 | 19 | 2.82 |  | |
|  | Right | 31 | -81 | 6 | 19 | 2.87 | 33 | -79 | 20 | 19 | 3.20 |  | |
| Inferior occipital gyrus | Left |  |  |  |  |  | -45 | -77 | -6 | 19 | 2.77 |  | |
|  | Right |  |  |  |  |  | 37 | -82 | -4 | 18 | 2.77 |  | |
| Cuneus | Left | -9 | -78 | 32 | 19 | 4.55 | -5 | -79 | 30 | 19 | 3.73 |  | |
|  | Right |  |  |  |  |  | 6 | -64 | 7 | 30 | 3.14 |  | |
| **Limbic structures** |  |  |  |  |  |  |  |  |  |  |  | Cognitive control and execution of voluntary movement through its connections with primary motor and premotor areas Spatial orientation through connections with posterior parietal cortex Scene recognition and spatial memory Somatomotor aspects of emotional and motivational states | |
| Anterior Cingulate | left |  |  |  |  |  | -3 | 33 | -9 | 32 | 2.65 |  | |
|  |  |  |  |  |  |  | -2 | 20 | 23 | 33 | 2.89 |  | |
|  | right | 9 | 32 | 14 | 24 | 3.10 | 8 | 34 | 14 | 32 | 2.68 |  | |
|  |  |  |  |  |  |  | 9 | 46 | 3 | 10 | 2.59 |  | |
| Posterior cingulate | left | -16 | -65 | 14 | 32 | 2.78 | -15 | -65 | 12 | 30 | 3.52 |  | |
|  | right |  |  |  |  |  | 19 | -65 | 17 | 31 | 2.55 |  | |
| Cingulate gyrus | left | -2 | 12 | 41 | 32 | 3.06 | -2 | -47 | 29 | 31 | 2.97 |  | |
|  |  |  |  |  |  |  | -2 | 14 | 26 | 24 | 2.77 |  | |
|  | right | 7 | 5 | 45 | 24 | 2.90 | 8 | 6 | 45 | 24 | 2.64 |  | |
| Parahippocampal gyrus | Left | -20 | -8 | -23 | 35 | 3.61 | -21 | -7 | -25 | 35 | 3.54 |  | |
|  | Right |  |  |  |  |  | 27 | -1 | -17 | A | 3.05 |  | |
|  |  |  |  |  |  |  | 20 | -31 | -3 | 27 | 2.67 |  | |
| **Insular cortex** |  |  |  |  |  |  |  |  |  |  |  | Connections with many areas of the cortex including sensory and motor areas and involved in sensation, vestibular function and stability Attention and set-shifting | |
|  | Left | -44 | 8 | 3 | 13 | 3.17 | -40 | -21 | -3 | 13 | 2.37 |  | |
|  | Right |  |  |  |  |  | 43 | -11 | -1 | 13 | 2.74 |  | |
|  |  |  |  |  |  |  |  |  |  |  |  |  | |
|  |  |  |  |  |  |  |  |  |  |  |  |  | |
| **Subcortical nuclei** |  |  |  |  |  |  |  |  |  |  |  |  | |
| Thalamus | Left |  |  |  |  |  | -4 | -15 | 15 | P | 2.5 | Relaying sensation, spatial sense and motor signals to and from the cerebral cortex | |
|  | right | 20 | -31 | 6 | P | 2.78 | 18 | -32 | 4 | P | 2.99 |  | |
| Claustrum | left |  |  |  |  |  | -29 | 10 | 8 |  | 3.60 | Integration of perceptual, cognitive and motor cortical functions | |
|  | right | 30 | 8 | 8 |  | 2.98 | 29 | 16 | 6 |  | 2.83 |  | |
| Putamen | Left |  |  |  |  |  | -21 | 8 | 4 |  | 2.74 | Modulation of motor activity | |
|  | Right | 26 | 10 | 7 |  | 2.77 | 22 | 9 | 5 |  | 2.91 |  | |
| Caudate nucleus (head) | left |  |  |  |  |  | -9 | 9 | 1 |  | 2.77 |  | |
|  | right |  |  |  |  |  | 5 | 13 | -3 |  |  |  | |
| **Cerebellum** |  |  |  |  |  |  |  |  |  |  |  | Coordination and postural control | |
| Anterior lobe | Left | -35 | -44 | -28 | C | 3.12 | -36 | -43 | -29 | C | 2.94 |  | |
|  | right | 27 | -52 | -26 | C | 3.11 | 8 | -55 | -15 | C | 3.26 |  | |
| Posterior lobe | left | -26 | -69 | -34 | Py | 3.72 | -22 | -71 | -33 | Py | 4.00 |  | |
|  |  | -13 | -65 | -18 | D | 2.79 | -24 | -59 | -21 | D | 3.18 |  | |
|  |  | -12 | -71 | -34 | U | 3.35 | -43 | -59 | -41 | Ct | 2.66 |  | |
|  |  | -39 | -39 | -38 | Ct | 2.80 | -16 | -71 | -32 | U | 3.76 |  | |
|  | right | 32 | -77 | -31 | Py | 3.28 | 28 | -68 | -33 | Py | 3.05 |  | |
|  |  | 30 | -65 | -15 | D | 2.98 | 20 | -65 | -22 | D | 2.63 |  | |
|  |  | 32 | -77 | -30 | T | 3.34 | 29 | -66 | -31 | Ct | 2.98 |  | |
|  |  |  |  |  |  |  | 11 | -71 | -34 | U | 3.57 |  | |

* False discovery thresholds for multiple comparisons at p=0.05: gait speed = 2.189, step length= 2.203; H=hemisphere; A=amgydala; P=pulvinar; C=culmen; Py=pyramis, D=declive; U=Uvula; Ct=cerebellar tonsil; T=tuber; Covariates in the models include age, sex, height, white matter hyperintensity volume and volume of normal appearing white matter; †Nieuwenhuys R, Voogd J, van Huijzen C. The human central nervous system, 4th ed. Berlin: Springer, 2008. The higher the t-value, the more significant the associations
